# A Window to the Brain: The Retina to Monitor the Progression and Efficacy of Saffron Repron^®^ Pre-Treatment in an LPS Model of Neuroinflammation and Memory Impairment

**DOI:** 10.3390/ph16091307

**Published:** 2023-09-15

**Authors:** Mattia Di Paolo, Francesca Corsi, Chiara Cerri, Silvia Bisti, Ilaria Piano, Claudia Gargini

**Affiliations:** 1 Department of Ophthalmology and Visual Science, University of Louisville, Louisville, KY 40202, USA; mattia.dipaolo@louisville.edu; 2 Istituto Nazionale di Biostrutture e Biosistemi (INBB), via Medaglie d’Oro 305, 00136 Roma, Italy; francesca.corsi@phd.unipi.it (F.C.); s.bisti@team.it (S.B.); maria.gargini@unipi.it (C.G.); 3 Department of Pharmacy, University of Pisa, Via Bonanno 6, 56126 Pisa, Italy; chiara.cerri@unipi.it; 4 Interdepartmental Research Center “Nutraceuticals and Food for Health”, University of Pisa, 56126 Pisa, Italy

**Keywords:** Alzheimer’s disease, retina, neuroinflammation, saffron Repron^®^

## Abstract

A mechanism shared by most neurodegenerative diseases, like Alzheimer’s disease (AD) and Parkinson’s disease (PD), is neuroinflammation. It has been shown to have a link between cognitive impairment and retinal function under neuroinflammatory conditions, confirming the essential role of the retina as a window to the brain. Here, we characterize a mouse model of LPS-induced neuroinflammation describing the parallel deterioration of both memory and visual function. Then, we demonstrate, using the Novel Object Recognition test (NOR) and electroretinogram (ERG) recordings, that preventive, chronic treatment with saffron Repron^®^ is able to reduce the neuroinflammation process and prevent the impairment of both cognitive and visual function. The improvement in behavioral and visual function is confirmed by the pattern of expression of neuroinflammation-related genes and related proteins where pre-treatment with Repron^®^ saffron presents a positive modulation compared with that obtained in animals treated with LPS alone. These results hold for retinal tissue and partially in the brain, where it appears that the onset of damage was delayed. This trend underlines the critical role of the retina as a most sensitive portion of the central nervous system to LPS-induced damage and could be used as a “sensor” for the early detection of neurodegenerative diseases such as Alzheimer’s.

## 1. Introduction

Neuroinflammation is an important factor contributing to cognitive impairment and neurodegenerative disorders such as Alzheimer’s disease (AD), Parkinson’s disease (PD), Huntington’s disease, multiple sclerosis (MS), and amyotrophic lateral sclerosis [1].

In particular, AD represents a severe neurodegenerative disorder typical of the elderly population characterized by progressive and irreversible behavioral and cognitive decline. The typical pathophysiology of AD sees the progressive loss of neurofilament and synaptic proteins, which impair neuronal cytoarchitecture, and the appearance of amyloid-beta (Aβ)-like neurofibrillary tangles in the neocortex [2]. In the pathogenesis of AD, in addition to the accumulation of plaques consisting of Aβ and sphingolipids, hyperphosphorylation of tau protein, increased apoptotic processes, and reduced synaptic plasticity, there is also a strong neuroinflammatory component [3].

Similarly, severe neuroinflammation occurs in patients with PD. Although most of the cases are idiopathic, PD patients show aggregates (Lewy bodies) of mutated alpha-synuclein (α-syn) [4], which induces progressive dysfunction of dopaminergic neurons in the striatum [5]. The toxic effect of misfolded α-syn leads to neurodegenerative processes and inflammation.

A crucial role in the initiation and development of this neuroinflammatory component is played by resident macrophages of the brain, microglia. Although acute neuroinflammation results are beneficial in preserving the morpho-functional properties of the tissue, chronic neuroinflammation is always considered detrimental to nervous tissue. Therefore, the duration of inflammation itself may be crucial in leading to beneficial or harmful outcomes for the brain. Under physiological conditions, microglia mainly eliminate metabolites and toxic substances. When stimulated, microglia migrate to the injured site and remove cellular debris [6].

Microglia exist in the M1 phenotype that initiates pro-inflammatory cascades that are neurotoxic, while M2 microglia suppress inflammation and have neuroprotective effects. In the pathogenesis of AD, neuroinflammation is triggered by Aβ deposition, phosphorylation of tau, and subsequent formation of neurofibrillary tangles, which stimulate microglia to differentiate toward the M1 phenotype; this phenomenon increases the M1/M2 ratio, which promotes cellular stress, neuronal dysfunction, and neurodegeneration [7]. Similarly, in PD, the M1/M2 ratio is increased by releasing soluble mediators, such as α-syn, from dying neurons [8]. From the activation of the M1 form, there is the production of inflammasomes and pro-inflammatory cytokines, which play a crucial role in the pathogenesis of AD [9] and PD [10], mediated mainly by the production of IL-1, IL-6, and TNF-α, complement activation, and the overproduction of reactive oxygen species [11,12].

In these diseases, as a consequence of the progressive atrophy of brain structures, including the different lobes (frontal, temporal, and parietal), entorhinal cortex, basal ganglia, cerebellum, and hippocampus, there is a reduction in cortical cognitive functions, such as memory, motor, and language functions, which may promote and/or exacerbate depression or anxiety states [13,14,15]. To date, there are no effective treatments to slow the progression of these diseases and/or their onset. In addition, the lack of an early diagnostic test to accurately determine the onset of AD or PD makes available treatments nearly ineffective because they are taken when neuronal damage is now irreversible [16,17]. Nowadays, the diagnosis of this type of neurodegeneration is based on clinical criteria derived from the performance of cognitive and functional tests such as the Mini-Mental Status Examination (MMSE) and Clinical Dementia Rating (CDR). Mnemonic TRAP [18] or neuroimaging techniques such as computed tomography (CT), magnetic resonance imaging (MRI), positron emission tomography (PET), and biomarker analysis in cerebrospinal fluid (CSF) are used [19]. To succeed in a preventive or effective treatment in patients to improve their quality of life, early diagnosis becomes imperative. Therefore, it is important to identify neurodegenerative biomarkers that are predictive of cognitive decline. In this regard, ophthalmologic evaluations have detected various ocular changes in patients with CNS disorders. In many of these disorders, ocular manifestations often precede brain symptoms, suggesting that ophthalmologic examinations could offer an early diagnosis of the underlying disease. Therefore, because the eye constitutes an extension of the brain, the evaluation of early ocular defects becomes an essential element in biomedical research [20,21,22]. In the retina, photoreceptors consist of rods and cones, which are essential for vision because they absorb photons and initiate downstream signal transmission. Rods and cones are responsible for imaging vision, including night and color vision. There is evidence that degeneration of photoreceptors leads to loss of vision and, eventually, irreversible blindness [23]. Despite the relevance of rods and cones in vision, abnormalities of these photoreceptors are largely unclear in AD. Here, we correlate the accumulation of Aβ plaques with alteration in photoreceptor function by measuring the progressive functional change of rods and cones in a model of induced neuroinflammation. We recorded scotopic and photopic electroretinogram (ERG) “in vivo” in control and degenerating animals.

In this context, our study suggests using the retina to monitor cognitive impairment in an animal model of lipopolysaccharide (LPS)-induced neuroinflammation in the C57Bl/6J mouse [24] to separate the inflammatory component of the pathology from other components, such as genetics. Oral pre-treatment with saffron, which has been known for centuries for its therapeutic properties [25,26,27], was performed in the same animal model. Specifically, we supplemented a selected type of saffron, named Repron^®^ (patent n° WO2015145316A1), which is characterized by a critical ratio among its chemical components. [28,29]. Strong data on the correlation between chemical compositions and neuroprotective efficacy have been obtained using several techniques in many experiments performed in both “in vitro” and “in vivo” models (see ref. [29]). Saffron has already been shown to be effective in patients with neurodegenerative retinal diseases such as age-related degenerative maculopathy (AMD) and Stargardt disease [30,31,32,33,34,35,36]. Here, we demonstrate the efficacy of Repron^®^ saffron in preventing neuroinflammatory damage, leading to good maintenance of cognitive and visual functions.

## 2. Results

**Figure 1:**
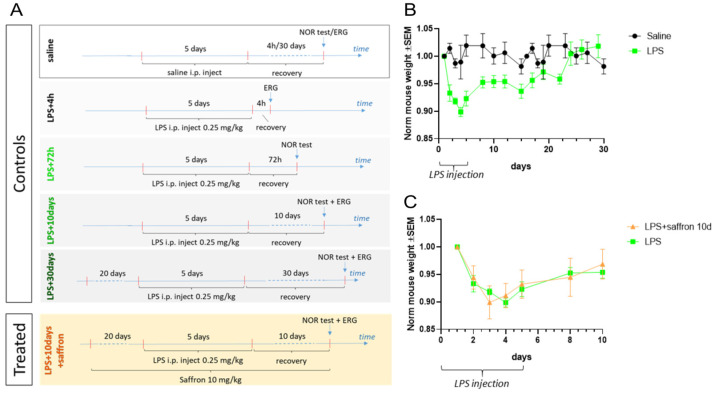
Schematic overview of the experimental design and weight monitoring during treatment with LPS and/or saffron. (**A**) Six-month-old male C57Bl/6J mice were randomly assigned to the different experimental groups: saline (*n* = 5); LPS + 4 h recovery (*n* = 9); LPS + 72 h recovery (*n* = 9); LPS + 10 days recovery (*n* = 9); LPS + 30 days recovery (*n* = 9); LPS + 10 days recovery + Repron^®^ saffron treatment (*n* = 11). (**B**) Body weight change in the saline group compared to the LPS group. (**C**) Body weight change in the LPS + 10 days recovery + Repron^®^ +saffron treatment group compared to the LPS group.

All animals entered into the experimental plan were monitored for behavioral and physiological changes (body weight monitoring) following LPS and saffron treatment. After LPS administration, mice showed classic signs of sickness, including decreased locomotion, hunched posture, as well as anorexia (Figure 1). As shown in the graph in Figure 1B, the LPS-treated animals showed a significant reduction in body weight, compared with the healthy control group, in the acute phase of LPS-induced inflammation corresponding to the 5-day administration period. Subsequently, losing about 10% of their mass in 3–4 days, the animals recovered body weight and returned to values superposable with those of the controls. Saffron treatment did not impact body weight changes in any way; in fact, in Figure 1C, it can be seen that the two curves always have a superimposable trend.

**Figure 2:**
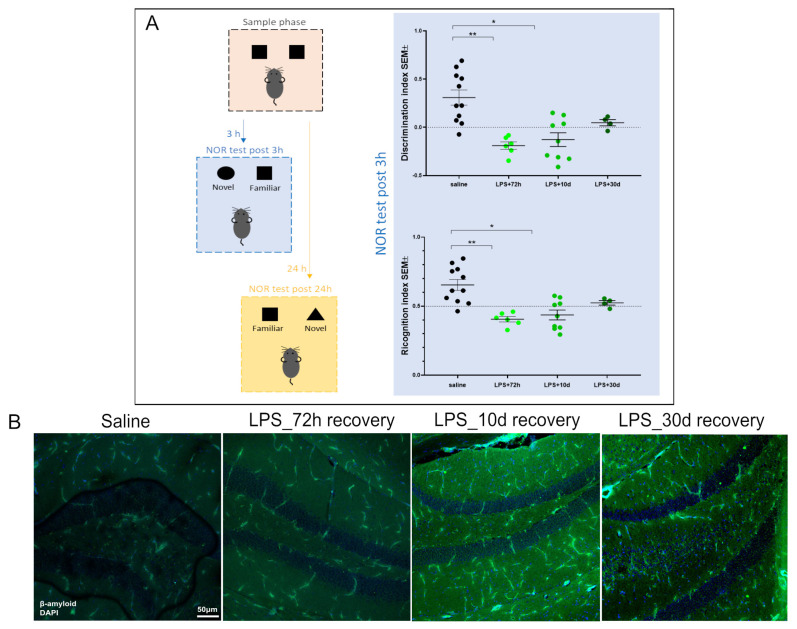
Effects of LPS treatment in the central nervous system. (**A**) Novel Object Recognition (NOR) test 3 h (blue) or 24 h (orange) in saline and LPS recovery groups (72 h, 10 d, and 30 d); saline (*n* = 5); LPS + 4 h recovery (*n* = 9); LPS + 72 h recovery (*n* = 9); LPS + 10 days recovery (*n* = 9); LPS + 30 days recovery (*n* = 9). The multiple experimental group means were analyzed by ordinary one-way ANOVA followed by a Bonferroni posthoc analysis. (* *p* < 0.05 saline vs. LPS + 10 days recovery; ** *p* < 0.01 saline vs. LPS 72 h recovery). (**B**) Representative brain images of animals treated with saline alone (ctrl group) and LPS at different recovery times, labeled against β−amyloid (green). Nuclei are labeled with DAPI (blue).

To confirm that LPS treatment was indeed capable of inducing inflammatory damage with physio-pathological features that could be traced to an Alzheimer-like model, the animals were subjected to behavioral assessments, and the tissues obtained from the same animals were analyzed by immunohistochemistry techniques (Figure 2). The panel in Figure 2A shows the results obtained by evaluation of the Discrimination index (DI) and Recognition index (RI) parameters obtained with the Novel Object recognition (NOR) test performed at 3 h post-sample phase at different post-LPS recovery time points (72 h, 10 and 30 days). As shown in the graphs in Figure 2A, both evaluated parameters undergo a significant reduction (DI < 0.0 and RI < 0.5) in the LPS-treated groups concerning the healthy control group at 72 h and 10 d post-LPS. After 30 d post-LPS, the animals showed a partial recovery of both indexes. Morphological analysis from the brain tissue immunostained for beta-amyloid highlights a higher presence of fibrillary accumulations at 10 d post-LPS, which correlates with the decline of cognitive abilities (Figure 2B).

**Figure 3:**
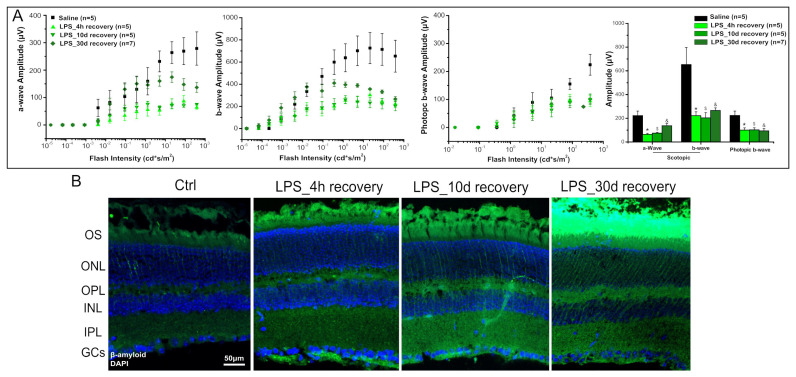
Effects of LPS treatment in the retina. (**A**) Scotopic a− and b−wave amplitude and photopic b−wave amplitude as a function of light stimulus intensity in the control group (black dots) and in the different post-LPS recovery times groups. Specifically, 4 h recovery (light green dots), 10 days’ recovery (green dots), and 30 days’ recovery (dark green dots). All statistical values are determined relative to the control group (black curve). The histogram shows the amplitude of scotopic a- and b-waves and photopic b-wave at the highest (377 cd*s/m^2^). Data are shown as MEAN ± SEM (*n* = 5, saline group, *n* = 5, 4 h recovery group, *n* = 5, 10 days’ recovery group, *n* = 7, 30 days’ recovery group); ANOVA one-way test. * *p* ≤ 0.05: LPS + 4 h recovery vs. saline group; $ *p* ≤ 0.05: LPS + 10 days recovery vs. saline group; & *p* ≤ 0.05: LPS + 30 days recovery vs. saline group. (**B**) Representative retina images of animals treated with saline alone (ctrl group) and LPS at different recovery times, labeled against β−amyloid (green). Nuclei are labeled DAPI (blue). Abbreviations: OS, outer segment; ONL, outer nuclear layer; OPL, outer plexiform layer; INL, inner nuclear layer; IPL, inner plexiform layer; GCs, ganglion cell layer.

To study possible correlations between brain and retinal damage, we also evaluated visual function with electroretinography. Figure 3A shows the results obtained by electroretinogram recording under both scotopic and photopic conditions at different recovery times (4 h, 10 d, and 30 d) post-LPS. At all time points, the amplitude of the characteristic ERG waves is significantly reduced compared to the control group that was treated with saline alone. The reduction in retinal neuron function corresponds to a parallel increase in beta-amyloid marking in the retina (Figure 3B).

**Figure 4:**
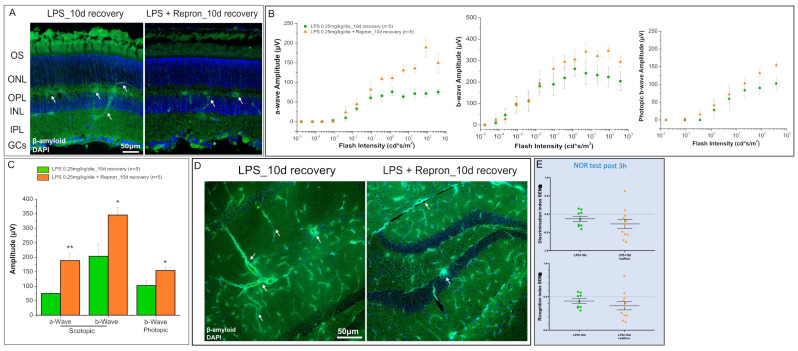
Efficacy of saffron Repron^®^ treatment after 10 days of recovery from LPS induction. (**A**) Representative retina images of animals group LPS + 10 days recovery and LPS + 10 days recovery + Repron^®^ saffron treatment group, labeled against β−amyloid (green). Nuclei are labeled DAPI (blue). Arrows indicate the accumulation of β−amyloid in the tissue. (**B**) Scotopic a− and b−wave amplitude and photopic b−wave amplitude as a function of light stimulus intensity in the LPS + 10 days recovery (*n* = 5, green dots) and the LPS + 10 days recovery + Repron^®^ saffron treatment group (*n* = 5, orange dots). (**C**) The histogram shows the amplitude of scotopic a- and b-waves and photopic b-wave at the highest (377 cd*s/m^2^). Data are shown as MEAN± SEM (SD) (*n* = 5, LPS + 10 days recovery group; *n* = 5, 10 LPS + 10 days recovery + Repron^®^ saffron treatment group); ANOVA one-way test. * *p* ≤ 0.05 scotopic and photopic b-wave LPS + 10 days recovery vs. LPS + 10 days recovery + Repron^®^; ** *p* ≤ 0.01 scotopic a-Wave LPS + 10 days recovery vs. LPS + 10 days recovery + Repron^®^. (**D**) Representative brain images of animals group LPS + 10 days recovery and LPS + 10 days recovery + Repron^®^ saffron treatment group, labeled against β−amyloid (green). Nuclei are labeled with DAPI (blue). Arrows indicate the accumulation of β−amyloid in the tissue. (**E**) Novel Object Recognition (NOR) test 3 h (blue) or 24 h (orange) in LPS + 10 days recovery and LPS + 10 days recovery + Repron^®^ saffron treatment groups.

Here, we established an animal model in which chronic LPS-induced inflammation was able to mimic cognitive impairment with characteristics reminiscent of those in an Alzheimer’s model. Furthermore, there was a parallelism between cognitive impairment and visual impairment, so we evaluated whether a preventive treatment with saffron Repron^®^ was able to counteract the processes leading to neurodegeneration. Figure 4A shows two retinal sections labeled for β-amyloid (in green), and it can be inferred that the labeling is less present in the retinal section obtained from animals previously treated with saffron Repron^®^, indicating less accumulation of these toxic aggregates for retinal nerve cells; the different levels of β-amyloid could be one of the effects leading to preservation of visual function that is significantly increased (Figure 4B,C), in the saffron-treated group compared with the control group (LPS), for both the scotopic and photopic ERG, indicating the improved function of both rods and cones. The effect at the brain level was also evaluated both morphologically and functionally. Similarly to retinal tissue, fibrillary accumulations of the labeled β-amyloid appear to be reduced in the brain section (Figure 4D); unfortunately, this reduction in toxic accumulations does not directly correlate with cognitive function assessed via the NOR test, which, as shown in Figure 4E, remains unchanged in the saffron Repron^®^-treated group compared to the relative control group. This functional difference could indicate a greater resilience ability of retinal tissue to trigger neuroprotective and recovery mechanisms that, overall, correspond to the preservation of visual function.

**Figure 5:**
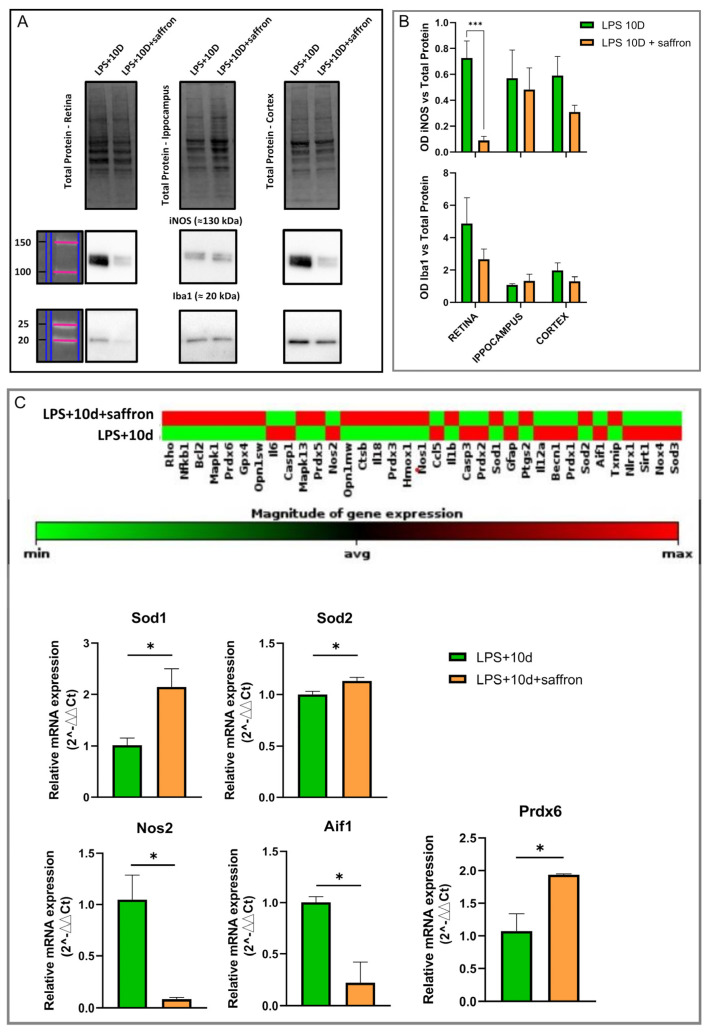
Genes expression (mRNAs) is involved in oxidative stress and inflammation processes in the retina. (**A**) Columns report the transcriptional expression levels of 35 genes in LPS + 10 days recovery and LPS + 10 days recovery + Repron^®^ saffron treatment groups. Each square represents the mean values of the values obtained after normalization on the mean expression of the housekeeping genes (GAPDH). The red color indicates the most up-regulated expression levels, while the green color indicates the least down-regulated expression levels. Below are bar graphs of significantly regulated genes extracted from the heatmap analysis. Bars represent mean ± SEM; statistic test: unpaired *t*-test, * *p* ≤ 0.05 LPS + 10 days recovery vs. LPS + 10 days recovery + Repron^®^. (**B**,**C**) Image of the bands related to Total Protein, iNOS, and Iba1. Histogram relative to the quantification, by optical densitometry, of iNOS and Iba1 in the different tissue and the two treatment groups. The number of animals used for WB analysis were LPS + 10 days recovery (*n* = 4) and LPS + 10 days recovery + Repron^®^ saffron treatment (*n* = 4). Bars represent mean ± SEM; statistic test: unpaired *t*-test, *** *p* ≤ 0.001 iNOS retina sample LPS + 10 days recovery vs. LPS + 10 days recovery + Repron^®^.

To understand which biological processes may be involved in the visually revealed functional recovery, real-time PCR analysis was carried out on genes belonging to or converging in two of the processes most involved in the progression of retinal degeneration, such as oxidative stress and inflammation. In Figure 5A, it is evident from the heat map that the gene profile of the group treated with Repron^®^ is completely inverted concerning the control group. Furthermore, it is evident that in the group treated with saffron, genes involved in cell protection are up-regulated, and pro-inflammatory and cellular stress genes are down-regulated. In the same panel, the bar graphs extrapolated from the heat map are shown for the genes that significantly modulated the results between the two groups. Among these, the up-regulated genes are Sod1, Sod2, and Prdx6, while the down-regulated genes include Nos2 and Aif1. To confirm the data obtained, real-time PCR was carried out to evaluate the levels of some related proteins. Figure 5B,C show, respectively, the examples of the membranes used for protein quantification and compared in the various tissues, and the bar graphs show how, at the retinal level, there is a significant reduction of the proteins whose genes were down-regulated (Iba1 and iNOS). From the same bars graph, it is also possible to note that the protein levels of these markers do not follow the same trend either in the cortex or in the hippocampus. This result could further confirm the hypothesis that the retina has a greater ability to respond to preventive and neuroprotective treatments than brain tissue. This greater sensitivity of the retinal tissue could be exploited as a monitoring tool for brain neurodegenerative pathologies such as Alzheimer’s.

## 3. Discussion

Data obtained in the present research project provide evidence that the visual function in LPS-treated mice is highly compromised and can be partly rescued by pre-treatment with saffron. This result might support the idea of testing vision to get an early sign of central cortical dysfunction. Consistent with previous studies, we found that mice injected with LPS showed signs of cognitive deficits and neuroinflammation through modulation of genes related to nuclear factor kappa B (NF-κB) signaling, which regulates microglia activation and pro-inflammatory cytokine release in the brain [37,38]. In addition, we have shown that the systemic LPS injection activates neuroinflammatory processes, leading to visual deficit [39]. This evidence adds to the ocular manifestations of brain neurodegenerative diseases, like AD, that have been extensively studied in recent decades. Neurological and retinal damage can be associated with neuroinflammation as well as with the accumulation of Aβ aggregates. The results obtained in our study show that, after LPS injections, there is a deficit at the retinal level, leading to a reduction in neuronal function due first to changes in cellular metabolism and then to the chronicization of inflammatory processes that persist even after the cessation of LPS administration, as well as to a progressive increase in Aβ aggregates at the level of the outer segments of photoreceptors, where the key elements of the phototransduction process are located. These results are consistent with what other authors have already shown regarding the pathological accumulation of Aβ and phosphorylated tau (p-tau), which are the typical signs of AD in the eyes of patients with AD and the eyes of transgenic mouse models of this disease. The accumulation of Aβ and p-tau in the eye has been associated with ocular damage, including cataract formation, loss of retinal neurons, RNFL thinning, and altered axonal growth. Further, retinal Aβ deposition is also a phenomenon that occurs with increasing age and was not limited to sub-RPE regions but was unexpectedly found to be accumulated in the outer segments of photoreceptors in aged mice. In such deposits, by 12 months, the outer segments were completely enveloped by a material containing Aβ [40]; this evidence suggests an impairment of the recycling mechanism of the photopigments contained in the outer segment of the photoreceptors and a consequent worsening of the retinal function as also occurs in ocular pathologies such as aged-macular degeneration (AMD). Overall, the prominent signs of AD in the eye underline the close relation between the retina and the brain [41,42].

In recent years, the use of natural substances for the prevention of neurodegenerative diseases has become one of the primary goals for improving the living conditions of these patients. Regarding natural substances, those with biological effects derived from polyphenols and flavonoids are some of the most represented therapeutic agents for slowing or preventing neurological disorders [43,44,45,46]. Among the foods with the highest nutraceutical potential, one of the oldest and most valuable spices that have been placed for centuries is saffron. Several studies have shown how the chemical composition of saffron is closely related to some bioactive properties of this spice. Chemical analysis of several saffron samples, conducted in parallel with the treatment of animal models, has shown that the neuroprotective activity of this spice depends on the content of some of its active compounds, particularly the concentration of the two most abundant crocins: trans-crocetin di-(β-D-gentiobiosyl) ester and trans-crocetin (β-D-gentiobiosyl) (β-D-glucosyl) ester. The threshold concentration values of the two crocins (17% mg/g and 8% mg/g, respectively) were identified. Saffron has less or no neuroprotective activity. These results led to the filing of an international patent, to which a saffron quality label (REPRON^®^) is attached [29,47]. In general, the relative percentage of bioactive compounds was found to be strongly dependent on the origin of the spice, with a general amount of 10 percent in dry saffron for crocins, 4 percent for picrocrocin, and safranal accounting for 70 percent of the volatile fraction. Interestingly, saffron (REPRON^®^) has been tested in neuronal culture stressed with beta-amyloid, and it has been reported the potentiality of saffron treatment for reversing Aβ-neurotoxicity and rescuing network-wide firing of neural stem cells (NSCs). Saffron activates a protective neuronal response to the stress induced by beta-amyloid to maintain neuronal function [48].

In the present study, in addition to confirming that chronic inflammation induced by LPS can lead to cognitive impairment typical of Alzheimer’s. We have also demonstrated that the retina undergoes functional modifications that slightly precede and follow in an overlapping manner the cognitive deficits visible both in the behavioral assay through a test for the evaluation of memory, such as NOR, and through one of the functional tests, also used in the clinic, for the evaluation of the activity of the retinal neurons such as the ERG. Translated to humans, where it is now known that AD patients show signs of retinal degeneration postmortem [49], it could be exploited in subjects familiar with AD to identify possible processes that preclude the onset of the disease with a simple outpatient evaluation of the ERG.

Last but not least, we have demonstrated that preventive treatment with Repron^®^ saffron can reverse the process of neuroinflammation by modulating the expression level of genes correlated with this pathway, also demonstrated by others [50]. And, as far as the retina is concerned, it is also able to preserve visual function. Unfortunately, this trend does not directly affect complex cerebral functions like object recognition; the cognitive deficits did not improve following the treatment with saffron. However, the fact that in the cerebral of the brain, the levels of pro-inflammatory markers decreased and anti-inflammatory markers increased suggests that the Repron^®^ saffron effect might be more evident by assessing earlier symptoms of brain cognitive decline, like anxiety. This difference in behavior between the retina and the brain could result in a greater ability of the retina to respond to external interventions by implementing resilience mechanisms that make it more sensitive to the treatments themselves [51]. A second possibility is that treatment has to be continued longer and at different dosages.

## 4. Materials and Methods

### 4.1. Mice

Wild-type C57Bl/6J mice (male, 6 months of age) were maintained under a 12 h light/12 h dark cycle with free access to water and food. Mice were treated according to Italian and European institutional guidelines, following experimental protocols approved by the Animal Welfare Organization (OPBA) of the University of Pisa and the Italian Ministry of Health (n° 353/2021-PR).

### 4.2. Mouse Model of Neuroinflammation and Saffron Repron^®^ Treatment

Mouse was randomly divided into six groups (saline, LPS + 4 h, LPS + 72 h, LPS + 10 days, LPS + 30 days, LPS + 10 days + saffron; *n* = 12 for each experimental group). LPS treatment was carried out at a dose of 0.25 mg/kg following a sub-chronic (5 days × 0.25 mg/kg) administration protocol [52]; functional tests were performed after 4 and 72 h and after 10 and 30 days after the induction of neurodegeneration with LPS (hereafter reported as recovery time from the end of LPS administration). Oral treatment with saffron Repron^®^ was given at a dose of 10 mg/kg/day in drinking water [28,29,30,32], starting from 20 days before the induction of damage with LPS until 10 days after the end of LPS treatment. The treatment protocol was designed to start earlier to compensate for the inevitable mild acute inflammation induced in the first LPS administration.

Among the recovery groups after LPS treatment, we chose to perform the treatment with saffron Repron^®^ in the LPS + 10 days group because, here, the signs of neuroinflammation are still clearly visible. The health status of the animals was also monitored throughout the trial through body weight assessment; no animals had more than 20% weight loss, which was defined as a human endpoint to prevent severe conditions (3Rs—replace, reduce, and refine—principles and a legal requirement in the European Union Directive 2010/63/EU). The schematization of treatment protocols and the results obtained for the body weight monitoring are shown in Figure 1.

### 4.3. Novel Object Recognition (NOR) Test

The novel object recognition (NOR) test is based on the natural propensity of rodents to investigate a novel object and to explore new objects when they are presented with a novel environment; it is used to evaluate both short-term and long-term memory. Animals, during the 5-day treatment period and 4 h after LPS injection, were individually habituated to an open field for 10 min in the absence of objects. Two identical objects were placed in the arena, and the mouse was left free to explore the objects for five minutes (sample phase). For each animal, this phase was repeated 3 times with an interval of 15 min. After each exploration, the animal was gently placed back inside its cage with water and food available [53]. The test phase for memory assessment began 3 h after the end of the sample phase: for this phase, one of the two identical objects used in the “sample phase” was replaced with a new object for the mouse. The animal was gently placed inside the arena and was left free to explore the area for 5 min. A camera recorded the mouse’s behavior. At the end of the experiment, the animal was placed back in its cage. The NOR test videos were analyzed with open-access tracking software (ToxTrac v2.98), from which the mobility rate, distance, and trajectories traveled by each animal were extracted. Then, they were processed through image analysis software (ImageJ-win32) to quantify trajectories in peripheral areas and central areas of the arena [53]. The data obtained were statistically tested and represented in graphs using analysis software (GraphPad Prism 8).

The Discrimination Index (DI) was calculated as follows: DI = (time spent on exploring novel object—time spent on exploring old object)/(time spent on exploring novel object + time spent on exploring old object).

The Recognition Index (RI) was calculated as follows: RI = time spent on the new object/(time spent on the new object + time spent on the familiar object).

The two-index analysis gives information about the behavior of animals according to their ability to remember objects:

- DI = 0 and RI = 0.5: Time (T) new object exploration = T familiar object exploration; the animal does not remember.
- DI > 0 and RI > 0.5: T new object exploration > T familiar object exploration, the animal remembers.
- DI < 0 and RI < 0.5: T new object exploration < T familiar object exploration, the animal does not remember or remembers and is afraid of the new object (is anxious/depressed).

### 4.4. Electroretinogram (ERG)

The general procedure for animal preparation, anesthesia (intraperitoneal injection (i.p.) of 20% urethane in a saline buffer (0.9% NaCl) at a dose of 0.1 mL/10 g body weight), ERG recording, light stimulation, and data analysis has been described in detail previously [34]. Briefly, ERGs were recorded in the dark through coiled gold electrodes in close touch with the cornea, hydrated by a thin layer of gel (Lacrinom, Farmigea), while the reference (ground) electrode was placed at the level of the scalp. The animal—after being put inside the 30 cm diameter Ganzfeld sphere, the inner surface of which was coated with white reflective paint—was exposed to light stimulation; the latter was carried out with a white-light electric flash (SUNPACK B3600 DX, Tecad Company, Tokyo, Japan), and six calibrated neutral density filters were used to modulate the intensity. For the scotopic ERG recordings, mice were subjected to a single flash of increasing intensity (from 1.71 × 10^−5^ to 377 cd*s/m^2^, in increments of 0.6 log units), each repeated six times, with the interval between stimuli varying from 20 s for dim flashes to 45 s for brighter flashes. Isolated cone (photopic) components were obtained by overlapping flashes from 0.016 to 377 cd*s/m^2^ with a constant background of saturating intensity for the rods (30 cd/m^2^) after at least 15 min of background alone. The amplitude of the scotopic a-wave was measured at 7 ms from the onset of the light stimulus, and the b-wave was measured from the peak of the a-wave to the peak of the b-wave. The amplitude of the photopic b-wave was measured from the baseline to the peak of the b-wave. The data were analyzed with the LabVIEW 2019 program (National Instruments, Austin, TX, USA).

### 4.5. Real-Time Polymerase Chain Reaction (PCR) 

**Scheme 1:**
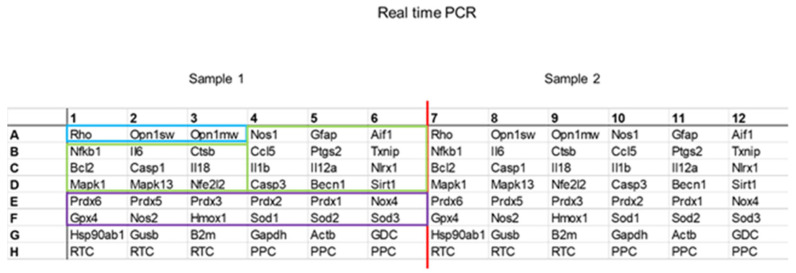
Cyan: photoreceptor-specific genes; Green: genes involved in inflammatory processes; Purple: genes involved in oxidative stress; Rows G/H: housekeeping and control genes.

The purification and extraction of total RNA from retina tissues were performed by miRNeasy Micro Kit (Qiagen, Hilden, Germany). The extracted RNA was quantified by NanoDrop Lite spectrophotometer (ThermoFisher Scientific, Nanodrop Technologies, Wilmington, DE, USA) and retro-transcribed by RT2 First Strand Kit (Qiagen, Hilden, Germany). The obtained cDNA was used for analysis by real-time PCR of multiple genes, using RT2 Profiler PCR Array Custom (Scheme 1) (#CLAM41859, Qiagen, Hilden, Germany). Expression analysis was performed through the Gene Globe Data Analysis Center (https://geneglobe.qiagen.com (accessed on 4 May 2023); Qiagen, Hilden, Germany). The normalized expression value is expressed as fold-change relative to the control group (saline-treated animals).

### 4.6. Western Blot

Retinal tissues were lysed with modified RIPA buffer, described by [54], while the brain and hippocampus were lysed with RIPA buffer (150 mM NaCl, 50 mM Tris-HCl pH 8, 1% Igepal, 0.5% Na-deoxycholate, 0.1% SDS and protease inhibitors, 1 µM Orthovanadate, and 0.1 mg/mL PMSF), and proteins quantified with a Bradford assay. The procedure of electrophoretic run of protein samples, antibodies incubation, and analysis was described previously in [55]. Briefly, 25 μg of each cell protein extract was mixed with the Laemmli 2X solution and resolved by electrophoresis SDS-PAGE, supported by the use of precast stain-free gel, and after activation, the separated proteins were transferred to PVDF membranes. The membranes were incubated with the antibody in Table 1. Analysis of the densitometry was undertaken using Bio-Rad ImageLab software 6.0.

### 4.7. Immunohistochemistry

Tissue (brain and eyes) for immunohistochemistry were collected immediately after animal sacrifice. The eyes were fixed in 4% paraformaldehyde for 45 min and dissected for removal of the cornea and lens. Afterward, the eyes were cryo-protected by a solution of sucrose at 30%, included in OCT Tissue Teck, and stored at −80 °C. In parallel, isolated cerebral hemispheres underwent a similar process without dissections but with a longer fixation time (3 days). Cryosections of each tissue were obtained using a Cryostat (Leyca) and collected on object slides (10.3389/fnsys.2014.00099). After a few washes with phosphate-buffered saline (PBS), the sections were subjected to saturation of nonspecific bonds by bovine serum solution (BSA) for about 1 h. Next, the tissues were incubated at 4 °C, with the appropriate concentrations of the primary antibodies of interest (Table 1). Retina samples were incubated overnight with the primary antibody in 1% BSA; while brain samples were incubated for two days with the primary antibody in 5% BSA. After a few washes with PBS, the relevant fluorescent secondary antibody was incubated for 2 h at room temperature. Finally, the tissues were labeled with a nuclear intercalant (DAPI) and included in glycerol gelatin with a coverslip. Representative images of the retina, hippocampus, and cortex were acquired through a confocal microscope (Nikon) and processed with analysis software (ImageJ).

### 4.8. Statistic

Data have been analyzed using GraphPad Prism Software or OrigiLab 8.2. According to the normality test outcome, a correspondent parametric or not parametric test has been performed. Corresponding details about statistical tests are reported in the legend of each figure.

## 5. Conclusions

Overall, the results described in the present work confirm the fundamental role of neuroinflammation in the progression of neurodegenerative pathologies and open new perspectives for the development of early diagnoses using the eye and the significant changes in visual function as a tool. Furthermore, the evidence that preventive treatment with Repron saffron might preserve cognitive and visual functions by modulating neuroinflammatory processes through gene and protein regulation makes this treatment very interesting. Further experiments are necessary to provide a more complete knowledge about long-term results and ways of action. Nevertheless, given the high safety and the almost total absence of toxic effects, the administration might be started in those subjects who, although not yet showing symptoms, are more predisposed to the development of the disease according to a variety of criteria.

## Figures and Tables

**Table 1:** List of antibodies for Western blotting and immunohistochemistry.

Antibody	Host	Company	Work Dilution	Application
Anti-Beta-Amyloid 1–42	Rabbit	Sigma-Aldrich, St. Louis, MI, USA AB5078P	1:500	IHC
Anti-Iba1	Mouse	Millipore, Temecula, CA, USA MABN92	1:1000	WB
Anti-iNOS	Mouse	R&D Systems, Minneapolis, MN, USA MAB9502	1:500	WB
Anti-rabbit Alexa Flour-488		BioRad, Hercules, CA, USA	1:500	IHC
Anti-mouse IgG HRP conjugated		Sigma-Aldrich	1:5000	WB

## Data Availability

The experimental data that support the figures within this paper, and other findings from this study, are hosted at the Department of Pharmacy, University of Pisa and can be accessed by contacting the corresponding author.
